# Understanding AL amyloidosis with a little help from *in vivo* models

**DOI:** 10.3389/fimmu.2022.1008449

**Published:** 2022-11-15

**Authors:** Gemma Martinez-Rivas, Sébastien Bender, Christophe Sirac

**Affiliations:** Contrôle de la Réponse Immune B et des Lymphoproliférations, CNRS UMR 7276 INSERM UMR 1262, Université de Limoges, Limoges, France

**Keywords:** immunoglobulin light chain (LC), amyloidosis, animal models, toxicity, AL

## Abstract

Monoclonal immunoglobulin (Ig) light chain amyloidosis (AL) is a rare but severe disease that may occur when a B or plasma cell clone secretes an excess of free Ig light chains (LCs). Some of these LCs tend to aggregate into organized fibrils with a β-sheet structure, the so-called amyloid fibrils, and deposit into the extracellular compartment of organs, such as the heart or kidneys, causing their dysfunction. Recent findings have confirmed that the core of the amyloid fibrils is constituted by the variable (V) domain of the LCs, but the mechanisms underlying the unfolding and aggregation of this fragment and its deposition are still unclear. Moreover, in addition to the mechanical constraints exerted by the massive accumulation of amyloid fibrils in organs, the direct toxicity of these variable domain LCs, full-length light chains, or primary amyloid precursors (oligomers) seems to play a role in the pathogenesis of the disease. Many *in vitro* studies have focused on these topics, but the variability of this disease, in which each LC presents unique properties, and the extent and complexity of affected organs make its study *in vivo* very difficult. Accordingly, several groups have focused on the development of animal models for years, with some encouraging but mostly disappointing results. In this review, we discuss the experimental models that have been used to better understand the unknowns of this pathology with an emphasis on *in vivo* approaches. We also focus on why reliable AL amyloidosis animal models remain so difficult to obtain and what this tells us about the pathophysiology of the disease.

## Introduction

Immunoglobulin light chain amyloidosis, or AL amyloidosis, is a hematological disease in which a plasma cell clone (or in rare situations a B cell clone) (1) produces a monoclonal immunoglobulin (Ig) light chain (LC), which aggregates into a characteristic organized structure, called amyloid fibrils (2). These amyloid fibrils accumulate in the extracellular compartment of almost every organ such as the kidneys and the heart (75%), leading *in fine* to their dysfunction. The general steps leading to the formation of amyloid fibrils have been well characterized *in vitro*, starting with a lag phase during which misfolded LCs slowly aggregate to form non-fibrillar oligomers followed by the first fibrils nuclei and protofibrils (2–4). When sufficient seeding is reached, mature fibrils are rapidly formed by an autocatalytic process involving surrounding amyloidogenic LCs (2). However, the mechanisms leading to the initial misfolding of LCs and their structural conversion from a soluble globular to a cross β-pleated sheet flat structure are still poorly understood (5). One reason is the uniqueness of every pathogenic LC, making AL amyloidosis a heterogeneous disease that is difficult to study (6). *In vitro* biochemical and structural studies, cellular models, and, more recently, bioinformatic tools gave us some clues about the unknowns of this disease and will be discussed in this review. However, these techniques reproduce only partially the *in vivo* context of this pathology, leaving aside the complex interactions and environment of the LCs in tissues or circulation. However, the recent improvements in patients’ amyloid deposit analyses through mass spectrometry and cryogenic electron microscopy (Cryo-EM) significantly advance our knowledge of AL amyloidosis. But they also raised new questions about how and why some LCs form fibrils and highlighted a new layer of complexity and heterogeneity in AL amyloid structures and composition. This review focuses on the difficulties of accurately modeling such a complex disease and how some discrepancies between *in vitro* experiments, *in vivo* models, and clinical observations could help in understanding the pathophysiology of AL amyloidosis.

## To V or not to V?

The variable (V) and constant (C) domains, which are two tandem Ig domains, compose LCs. As its name suggests, the V domain alone bears the variability of the LCs. This variability is due to the imprecise rearrangements between the variable (V) and the joining (J) gene segments to form the V domain exon and the subsequent somatic hypermutations during the antigenic response (7), which makes each LC a unique protein. However, amyloid deposition has been mainly associated with a few V domains (hereafter called VL), such as gene segments *IGLV6-57*, *IGLV2-14*, *IGLV1-44*, or *IGKV1-33*, and each VL seems to have a tropism for one or more organs (8–14). For these reasons, the VL has been suspected for years to be responsible for amyloid fibril formation in patients, coherently with the first amino acid sequencing of AL fibrils (15, 16). Accordingly, many crucial studies showed that mutations in the VL are responsible for the destabilization of the protein leading to the structural conversion from a soluble globular to a stacked flat structure. Consequently, many laboratories working on the process of AL fibrillogenesis have focused their attention on these domains, often working with isolated recombinant VLs (10–12). However, despite the multiple LC sequences available in the literature (9, 13, 14) or databases like AL base (https://albase.bumc.bu.edu), it is still impossible to predict the amyloidogenicity of an LC solely based on its amino acid sequence since each LC presents with multiple combinations of mutations that can account for their aggregation propensity. Several predictive tools have been developed based on the physicochemical properties of protein sequences (17–19) and, more recently, have been systematized using machine learning approaches (20, 21), but they still fail to predict all amyloid LC sequences. They also deserve to be tested with other aggregation prone LCs (from light chain deposition diseases, light chain proximal tubulopathy with crystals, etc.) to determine if they predict specifically amyloidogenicity or just instability of the LCs. Consequently, we cannot list in this review all the mutations suspected to participate in their amyloidogenicity, but we can try to draw a general outline of the knowledge so far based on these *in vitro* studies: 1) AL LCs can acquire a misfolded structure due to mutations in the VL affecting their stability, their dynamic properties, and their susceptibility to proteolysis (10, 22–27). 2) VLs are more prone to form fibrils *in vitro* than their full-length counterparts (26, 28, 29), at least under physiological conditions (37°C and pH ~7) (28, 30, 31). 3) VL or full-length LC dimers protect from misfolding and proteolysis, and mutations affecting the dimer stability seem to be involved in fibril formation (11, 12, 28), leading to the development of LC dimer stabilizers to slow the amyloid cascade (32, 33). If there is no debate about the role of the VL in amyloidogenesis of LCs, it does not mean that the C domain is useless. Accordingly, few biochemical studies showed that the full-length LCs could be involved in the initial nucleation thanks to its stability which serves as a platform for the subsequent elongation by unstable VLs (34–36). These experimental results are in line with the observations made by mass spectrometry both in diagnosis using laser microdissection/mass spectrometry (LMD/MS) (37) and in more careful analyses of deposited protein contents (38) showing that a complete or partially degraded C domain is always present in the amyloid deposits (39), and the degradation patterns argue for a post-deposition proteolysis. Whether C domain proteolysis occurs before or after aggregation and contributes or conversely protects from amyloidogenesis *in vivo* is still under debate (3, 39), but using the sole VL to study amyloidogenesis of LCs *in vitro* is likely a reduced approach that do not mirror the *in vivo* process.

It is important to distinguish the amyloid fibril from the amyloid deposits. In recent years, the crucial contribution of the Cryo-EM demonstrated that the fibril core itself is only composed of the VL (full or partially degraded), and all the intermolecular interactions in the fibril are established by this domain (40, 41). As expected by the variability of their amino acid composition, each fibril structure is unique, adding a layer of complexity to the understanding of AL amyloidosis formation (42). It also means that the C domain could be considered as a by-product of the fibril whose non-organized structure favors its access to local proteases, while the packed structures of the fibrils are resistant. Overall, it is still impossible from these studies to resolve this egg-and-chicken problem. We cannot exclude that a partial degradation, sufficient to destabilize the VL, allows the initial nucleation, and then the full-length LC elongates the fibrils in a self-catalytic process (43). For instance, a short cleavage of a few amino acids in the C-terminal part of the C domain is sufficient to remove the cysteine involved in the dimerization of LCs. C domains sticking out of the core structure would then be further degraded. Whether it could play a role in stabilizing the fibrils, for example, by hijacking or protecting the fibrils from proteolysis in a similar way as other associated components of the amyloid deposits like serum amyloid P component (SAP), glycosaminoglycans (GAGs), or apolipoprotein E (ApoE) (43), remains an open question. Once again, all these associated components are known to be involved in *in vivo* amyloidogenesis, and the C domain could play a similar role but is not amyloidogenic *per se*.

## Amyloidogenic light chain: A toxic relationship with cells and tissues

Another crucial question about AL amyloidosis is understanding the origin of the toxicity for tissues in this disease. There are currently two non-mutually exclusive hypotheses: toxicity due to a mechanical constraint generated by the presence of fibrils in the organs (amyloid burden) and direct toxicity of the soluble or oligomeric LCs for cells that do not require any accumulation of Congo red-positive material (proteotoxicity). The mechanical constraints exerted by the massive accumulation of insoluble and semi-rigid aggregates in organs can easily explain typical clinical symptoms of the disease, especially in contractile tissue like the heart or in the glomeruli, the filtering units of the kidney. However, strong clinical data challenge the unique role of the amyloid burden, especially for cardiac involvement. Indeed, treatments leading to a significant decrease of the circulating LCs lead to a rapid improvement of cardiac biomarkers (especially NT-proBNP) without an apparent decrease in the amyloid burden in the heart (44). Several experimental studies conducted *in vitro* with cellular models are in line with this clinical observation. The most studied cellular models are cardiomyocytes and cardiac fibroblasts. The overall toxic effects of LC exposure observed on these cells are the increase in reactive oxygen species (ROS) (45, 46), lysosomal dysfunction and autophagy impairment (47), the activation of a non-canonical p38 MAPK pathway (48, 49), and morphological damage of mitochondria (50). This would lead to a lack of contractility of cardiomyocytes and activation of cell death mechanisms (45, 46, 48, 49) when compared to non-amyloidogenic LCs or, more interestingly, to non-cardiotropic AL LCs, highlighting a strong specificity of LC cytotoxicity. Strikingly, although renal involvement in AL amyloidosis is frequent, there are only a few studies dealing with the toxicity of soluble AL LCs for renal cells, and they mostly revealed phenotypic changes and extracellular matrix remodeling, which are likely part of the overall toxicity of glomerulopathic LCs (51). Studies from Guillermo Herrera’s group, essentially working on mesangial cells exposed to non-pathogenic or glomerulopathic purified LCs, showed that AL LCs induce changes toward a macrophage-like phenotype (52), while LCs from light chain deposition disease (LCDD), another monoclonal gammopathy of renal significance characterized by amorphous LC deposits, induce a myofibroblastic phenotype. Consequently, AL LCs would be extensively internalized in caveolae and transported to lysosomes where they meet the conditions for rapid amyloidogenesis (proteolysis, denaturation, and acidic pH). Amyloid aggregates would then be extruded from the cells to invade extracellular spaces (53). A similar process was also shown by the same group in vascular smooth muscle cells (54), further emphasizing the role of lysosomes in AL amyloidosis formation, and is consistent with previous observations in splenic macrophages during the formation of AA amyloidosis (55). Ultrastructural studies in patients’ biopsies should be carefully reconsidered to confirm the presence of amyloid fibrils in target cells.

The resulting question of AL LC proteotoxicity is how they exert their effects on cells and tissues. In the kidney, sortilin-related receptor (SORL1) was recently presented as the common receptor for glomerulopathic LCs on mesangial cells using mass spectrometry, immunofluorescence, and ultrastructural studies (51). Functional studies with SORL1 knock-out cells are needed to confirm the role of this receptor. However, the expression of this receptor comes after the exposure of mesangial cells to glomerulopathic LCs (51). Consequently, while SORL1 is likely involved in the phenotypical changes of mesangial cells and the late intracellular processing of the LCs, early molecular events at the origin of this phenomenon are still unknown. In cardiomyocytes, the interactions between AL LCs and cellular components are even more elusive. No membrane receptor for LCs has been identified so far, but a mechanism of macropinocytosis of soluble proteins seems to allow the internalization of LCs in lysosomal compartments of cardiomyocytes and cardiac fibroblasts (31, 56–58). Interaction of the LCs with intracellular components could then induce cellular dysfunction as shown by Lavatelli’s group (46, 50). Fibrils are also internalized by macropinocytosis, although to a lower extent, likely due to their size. Two interesting studies showed that the internalization efficacy is size-dependent, with VLs being better endocytosed than full-length LCs or fibrils (56, 58). In the same publication from Ramirez-Alvarado’s group, they also showed that fibrillar species are far more toxic for cardiomyocytes than soluble LCs (56). Their hypothesis is that the presence of fibrils around the cardiomyocytes inhibits the cell–cell contact, which results in a strong cytostatic effect (56) and other cellular dysfunctions and microenvironment remodeling (59), even at very low concentrations, undetectable by ThT staining (56). This points out the issue of what is really toxic for cells. Similarly to biochemical studies on amyloidogenesis, it is difficult to summarize the proteotoxicity of AL LCs since many different protocols (concentrations, LCs purified from urines, and recombinant LCs produced in bacteria or mammalian cells), amyloid species (VL, full-length LCs, oligomers, and fibrils), cell types (primary or immortalized cell cultures, fibroblasts or cardiomyocytes, etc.), and LC sequences (degree of amyloidogenicity and organ tropism) have been used. Despite these different experimental conditions, all these studies agree about the direct proteotoxic effect of the amyloid precursor other than mature fibrils such as soluble full-length LCs (50, 60), partially degraded LCs, VLs (61), or oligomers (56) on cardiac cells. This specific toxicity for cardiac cells and no other cells was demonstrated by Lavatelli et al., who used human dermal fibroblasts as controls (50). The molecular basis of this specificity is not known, but one possibility is that the different proteostatic networks of the various tissues may account for the different susceptibility to light chain toxicity. Another concern resulting from these studies is how we can be sure that soluble LCs do not aggregate during the experiments. Most of the LCs and VLs used are highly amyloidogenic and could form fibrils or, at least, oligomers during the experiments, which can already be toxic for cells but whose concentration is too low to be detected by any test (4, 56). However, although it could easily explain the toxicity of AL LCs compared to non-amyloidogenic ones, it could not fully explain the specific toxicity of cardiotropic LCs for the heart compared to other AL LCs that were elegantly demonstrated (46, 50).

**Figure 1:**
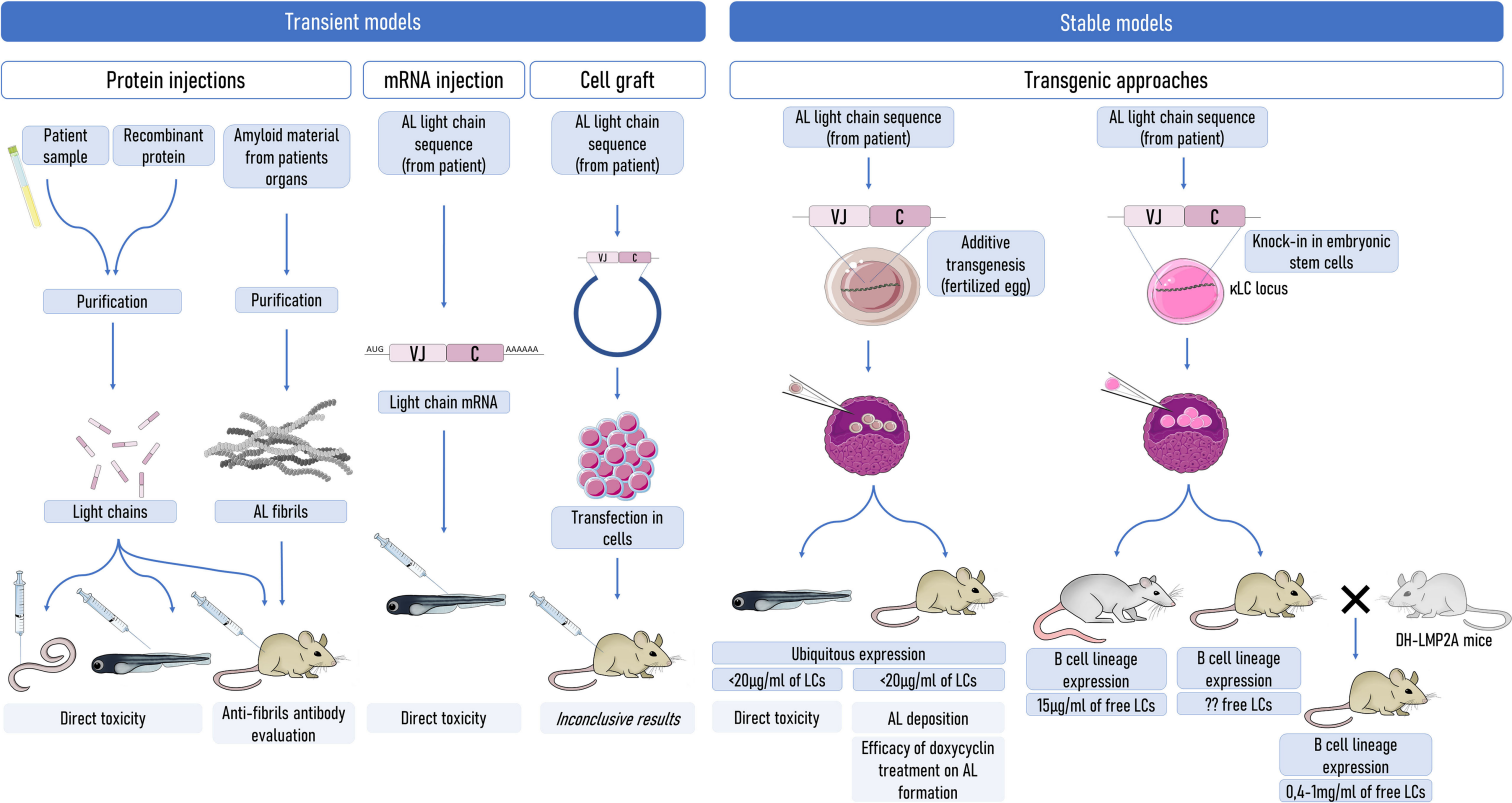
Strategies to model AL amyloidosis in animals. Transient models (left) were mostly used to evaluate the direct toxicity of amyloid LCs, but a few of them were also used to evaluate some therapeutics or amyloidogenesis. Stable production of the pathogenic LCs can be used to better mimic the pathology. However, the production of sufficient levels of circulating free LCs is an obstacle to obtaining spontaneous systemic amyloidogenesis. Even in mouse models with a production of free LCs at levels exceeding those in patients, amyloidogenesis does not occur spontaneously, highlighting a potential resistance of mice to AL amyloidogenesis. Understanding the factors that limit amyloidosis in mice could be of invaluable importance. LCs, light chains.

These *in vitro* experiments provided invaluable advances in the toxicity of the different LC species, but the diversity of experimental models used is a barrier to concluding on the mechanisms of toxicity of AL LCs. In addition, AL is a systemic and very complicated disease in which the diversity of LCs, the microenvironment in the tissues, and likely the specificity of each patient (6, 62) need to be taken into account. Further studies need to be carried out with more amyloidogenic LCs, VLs, and fibrils to establish the general toxicity of each type of protein species. Additionally, there is a need to develop *in vivo* models, reproducing as closely as possible the real pathophysiology of the disease. Some of them have already been used and their qualities and limits (**Figure 1**) are discussed below.

## *In vivo* surrogates: Modeling AL amyloidosis in non-mammal organisms

Simple organisms, such as drosophila, zebrafish, or the nematode *Caenorhabditis elegans*, have gained ground in biology because they have some advantages (costs, rapidity, and ethical concerns) over more developed models (e.g., rodents). Since several attempts have failed to create a reliable AL model until now, as will be discussed later in this review, *in vivo* studies have been carried out in these simple animal models. Because its pharynx, with autonomous contractile activity, is evolutionarily related to mammal hearts, *C. elegans* was used to study the cardiac toxicity of LCs. Accordingly, different purified or recombinant LCs from AL patients induced a diminution in the pharyngeal pumping ability of these animals compared to LCs from non-AL patients or AL patients with no cardiac involvement (60). This effect was associated with ROS-related oxidative damage, a reduced life span of the worms, and can be counteracted by antioxidant agents (60, 63). These animals did not display Congo red deposits, and the specific toxicity of cardiotropic LCs argues for direct toxicity of the LCs with the cautions cited above. In zebrafish, another *in vivo* model system to study AL LC toxicity, injections of 100 µg/ml of AL LCs also increased cardiac cell death resulting in fatal effects for the animals, with a median survival of 5 days after the LC injection (64). The injection of mRNA encoding for other AL LCs to zebrafish embryos showed similar effects, with an impairment of cardiac contractility and heart failure in more than 50% of injected animals (65). These seminal data encouraged Liao’s group to create a transgenic zebrafish model (66). Using a liver-specific expression, they obtained a production of about 125 µg/ml of an amyloidogenic LC, which is consistent with the level of FLC in newly diagnosed patients. They demonstrated a cytotoxic effect of the LCs for the cardiac cells with increased apoptosis and autophagy, but without affecting the general life span of the animals (66), which is in sharp contrast with the data obtained *in vitro* or with short animal exposure to AL LCs (64). They also observed increased proliferating cells in the heart of the transgenic zebrafish, which could explain that the cardiac toxicity of the AL LCs was counteracted by the regeneration of the tissue. This result reveals two limitations of this model, one specific to zebrafish and another with more general consequences. First, zebrafish, contrary to humans, have a striking capability of cardiac regeneration, being able to repair injured cardiac tissues (67). Consequently, the resistance to terminal cardiac dysfunction in the transgenic zebrafish would likely not have been observed in an animal model phylogenetically closer to humans. It remains difficult to determine whether the molecular and cellular toxicity of AL LCs in zebrafish fully mirrored that in human patients or whether they are specifically due to zebrafish peculiarities. Second, short exposure of LCs obtained with transient expression or the injection of a large amount of purified proteins is quite remote from the pathophysiology of AL amyloidosis, and the zebrafish model proved that it might induce different outcomes in animals and organs than the steady exposure to the protein observed in patients.

Finally, although these surrogate animal models are of invaluable interest to better understand the mechanisms of LC toxicity and possibly to evaluate new therapeutic approaches limiting this toxicity, none of them reproduces the cardinal feature of AL amyloidosis, the presence of Congo red-positive deposits. Consequently, they might not be fully considered models of AL amyloidosis since they reproduce only the invisible part of the disease and not the consequences of amyloid fibril accumulation on cellular toxicity and tissue remodeling. This is why many researchers have focused for years on the development of an AL animal model that could recapitulate all the typical features of this pathology, including amyloid fibril formation in organs similar to or close to human ones. Such models could help decipher the origin of the toxicity in this disease, and the earlier events resulting in the deposition of fibrils, and also test the efficacy and safety of new treatments that could improve patients’ outcomes. Mammals and more specifically rodents were the most studied models, but the difficulties in transposing *in vitro* observations to animals were revealed.

## Of mice and men: The complicated story of AL amyloidosis modeling in rodents

### In the line of sight: Attempts to create a rodent AL model

The first attempts to induce AL amyloidosis in mice were the “injection models” consisting of the intraperitoneal injection of grams of purified Bence–Jones proteins, which resulted in deposits in the blood vessel walls of mice’s kidneys (68, 69), but not in the glomeruli. This poorly physiological model was then abandoned for decades with few exceptions to evaluate the direct toxicity of AL LCs (48). The resurgence of this approach appeared when Herrera’s group used the penile vein for intravenous injections, which seem to offer an unsuspected advantage for LC delivery to kidneys. The injection of 1 mg per day of purified LCs from AL patients’ urines did induce AL fibril deposits in the glomeruli (53) and confirmed the *in vitro* observations of the group (see above) (52, 70). However, the main drawback of the injection method is the acute delivery of large quantities of LCs, which poorly reflects human pathology, usually characterized by a regular production of lower amounts of free LCs in serum (1). In addition, since mice can develop an immune response to human proteins, studies using repeated protein injections for more than 10 days should be considered with caution (71, 72). Other models were generated by injecting the so-called “amyloidomas”, which correspond to amyloid material purified from AL patients, subcutaneously in mice (72). This creates a mass of amyloidosis under the mice’s skin. This model cannot be used to study the pathogenesis of the disease, and its use in AL amyloidosis is limited to therapeutic or imaging molecule-binding assays, including anti-fibril antibodies (72–75). Some of them have been tested later in clinical trials (76–78) validating the contribution of the amyloidoma model in AL amyloidosis. Tumor graft models using monoclonal pathogenic LC-producing cell lines have been successful in reproducing in mice the initial lesions of monoclonal gammopathy of renal significance (MGRS) like LCDD or light chain proximal tubulopathy (LCTP) (79, 80). In contrast, this method never resulted in AL amyloid deposits in our experiments despite multiple attempts with different AL LCs. To our knowledge, other groups have developed AL LC-expressing cell lines, which also failed to reproduce AL amyloidosis in graft models (81–83). The often-given reason is the high proliferating properties of these cells that do not allow a sufficient accumulation of the pathogenic LCs before the sacrifice of animals due to tumor invasion. However, the fact that other LC-induced pathologies, including some requiring high quantities of monoclonal LCs (i.e., myeloma cast nephropathy) (80), were efficiently reproduced using tumor grafts refutes this sole explanation.

To avoid transient production of the pathogenic LCs that do not mirror human disease, transgenic approaches have also been employed to create reliable and more physiological AL amyloidosis models. Ward et al. (84) developed transgenic mice expressing an amyloidogenic λ6 LC under the control of the ubiquitous cytomegalovirus promoter. These mice were the first to develop few AL deposits in the lumen of the gastric glands of the stomach, probably due to the local expression of the LCs in an acidic environment, favoring their unfolding and fibrillogenesis. Levels of circulating human LCs were estimated at 5–10 µg/ml by Western blotting, and no deposit was detected in any other organs. Once again, although this model did not reflect the pathogenesis of AL amyloidosis, it was sufficient to demonstrate that doxycycline treatment inhibited amyloid formation *in vivo*, and it was recently useful to demonstrate the binding of a therapeutic molecule to AL fibrils (85). Interestingly, no sign of cardiac or renal toxicity due to the circulating LCs was found in these mice. Another transgenic model was generated by Nuvolone et al. (86), in which a human λ LC was conditionally expressed in all tissues (heart, kidney, lung, etc.). None of these mice developed AL amyloidosis in any organ or demonstrated any sign of toxicity.

All of these models seem to indicate that the level of expression of the free LCs likely plays a more important role in amyloidogenesis than previously thought. So far, none of the transgenic models reached the level of circulating free LCs needed to initiate systemic amyloid deposits.

### Another brick in the wall: Strategies to increase the expression of the pathogenic light chains in transgenic mice

**Figure 2:**
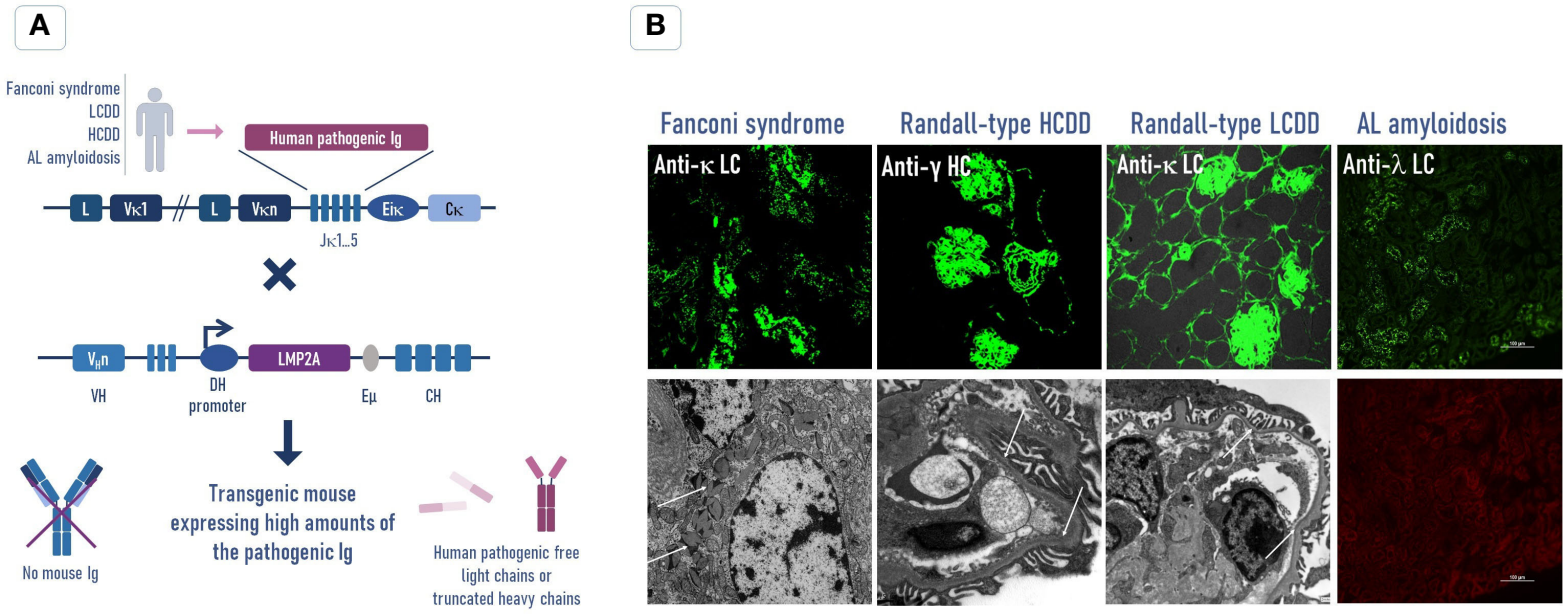
Transgenic strategy to increase the free pathogenic Ig production in mouse models to reproduce human Ig-related deposition diseases. **(A)** The human pathogenic Ig (light chain or truncated heavy chain) sequence was obtained from patients with an Ig-related deposition disease (Fanconi syndrome, LCDD, HCDD, or AL amyloidosis) and inserted by knock-in into the Ig kappa locus of mice. These mice were crossed with the DH-LMP2A strain mice, which present a normal B-cell development without producing Ig heavy chains, to avoid the association between the human pathogenic LC or HC and to increase its circulating concentration in transgenic mice. **(B)** Immunofluorescence (top) and electron microscopy (bottom) analyses of kidney sections show that the levels of circulating free LCs in these mice are sufficient to reproduce the main pathological features of human diseases. In the AL model, we did not observe Congo red amyloid deposits in the kidney (bottom) despite the presence of numerous human LC reabsorbed tubular cells (top). No amyloidosis was observed in any other organs (not shown). LCDD, light chain deposition disease; HCDD, heavy chain deposition disease; LC, light chain; HC, heavy chain.

In contrast to the transgenic models presented above, prioritizing a ubiquitous or liver-specific expression of the transgenic LCs, our group developed almost exclusively B cell-specific strategies. Plasma cells (PCs) represent the last stage of B-cell development and are mainly characterized by their capacity of secreting large quantities of antibodies. They are Ig factories able to secrete up to 10,000 Ig per second and can secrete antibodies for years. Consequently, we considered that they are the perfect cells to produce and secrete transgenic Ig and that the Ig loci bear all the necessary transcriptional enhancers to allow the best transcription of our transgenes. We developed in the late 1990s a B cell-specific transgenic mouse model using Ig transcriptional enhancers and promoters to express a κ LC from a patient with AL amyloidosis (unpublished results). Despite a significant production of the human LCs in serum (~1 mg/ml), we did not observe any amyloidosis in these mice or signs of morbidity throughout their life. In fact, most of the human LCs were associated with mouse heavy chains, strongly limiting the circulation of the pathogenic free LCs in serum, an issue that we recently solved (see below). More recently, we adopted a knock-in strategy in the mouse Ig kappa locus to develop several models of monoclonal Ig-related deposition diseases (87–90). The first one was a model of LC-induced Fanconi syndrome, in which the exon coding a VL from a patient was inserted to replace the joining (J) segments of the kappa locus (87) (**Figure 2**). During transcription, the human VL exon was naturally spliced to the mouse C domain exon, forming a complete human/mouse hybrid LC. The κ LCs in mice were expressed in over 90% of plasma cells closely mimicking the hematologic features of monoclonal gammopathies. These mice presented circulating LCs between 5 and 8 mg/ml. Even if LCs were mainly associated with the heavy chains (HCs), the concentration of free LCs was sufficient to reproduce crystal deposits in proximal tubular cells and typical tubular dysfunction associated with human disease. This model also provided proof that the VL is the only domain of the LCs involved in the pathogenesis of LC-induced Fanconi syndrome. Since rats appear as a suitable alternative to mice, as they are more sensitive to pathogenic lesions, sclerosis, and organ failure (91), we created a transgenic rat by the insertion in the rat immunoglobulin kappa locus of a human κ LC gene from an AL patient with strong kidney deposits and low circulating FLC (~100 mg/L). This LC was expressed in most B and plasma cells and circulated in blood at high concentrations (~1 mg/ml) but, similar to our first transgenic mice, most of the LCs were associated with rat heavy chains. In contrast with the LC-induced Fanconi model, the level of free LCs appeared to limit the trigger of amyloid deposits in the rats (92). The toxicity of LCs was also studied in the kidneys of these animals by measuring the creatinine level and the glomerulus histology, and none of the rats showed any sign of renal dysfunction or other morbidities due to the LC. To increase the proportion of free LCs in our transgenic models, we took advantage of the DH-LMP2A model, previously characterized by Casola et al. (93) (**Figure 2A**). To study the role of the Epstein–Barr virus latent membrane protein 2A (LMP2A) in infected B cells, they replaced the JH segments in the IgH locus with the gene encoding LMP2A. LMP2A effectively led to complete B-cell development in the absence of immunoglobulin heavy chains. We further showed that LMP2A mice also presented a striking accumulation of plasma cells, making them the ideal model for the production of large amounts of free LCs without any hematological disorder that could reduce the lifespan of mice (94). We first used this model to backcross with transgenic mice carrying a complete heavy chain from a patient with heavy chain deposition disease (HCDD) (88) and more recently with mice expressing a κ VL domain from a patient with LCDD (90). The homozygous strain produced only the human pathogenic κ free LCs, reaching ~1 mg/ml in the serum, which is similar to the level observed in LCDD patients (95). This model recapitulated the kidney lesions and dysfunctions of LCDD patients and proved to be a reliable platform for therapeutic investigations (**Figure 2B**). Additionally, we showed that plasma cells producing the pathogenic LC activated specific transcriptional pathways associated with endoplasmic reticulum stress associated with higher sensitivity to proteasome inhibitors. This model thus participated in the burgeoning field of research showing that abnormal monoclonal Igs may interfere with plasma cell fate and response to treatments (83).

**Figure 3:**
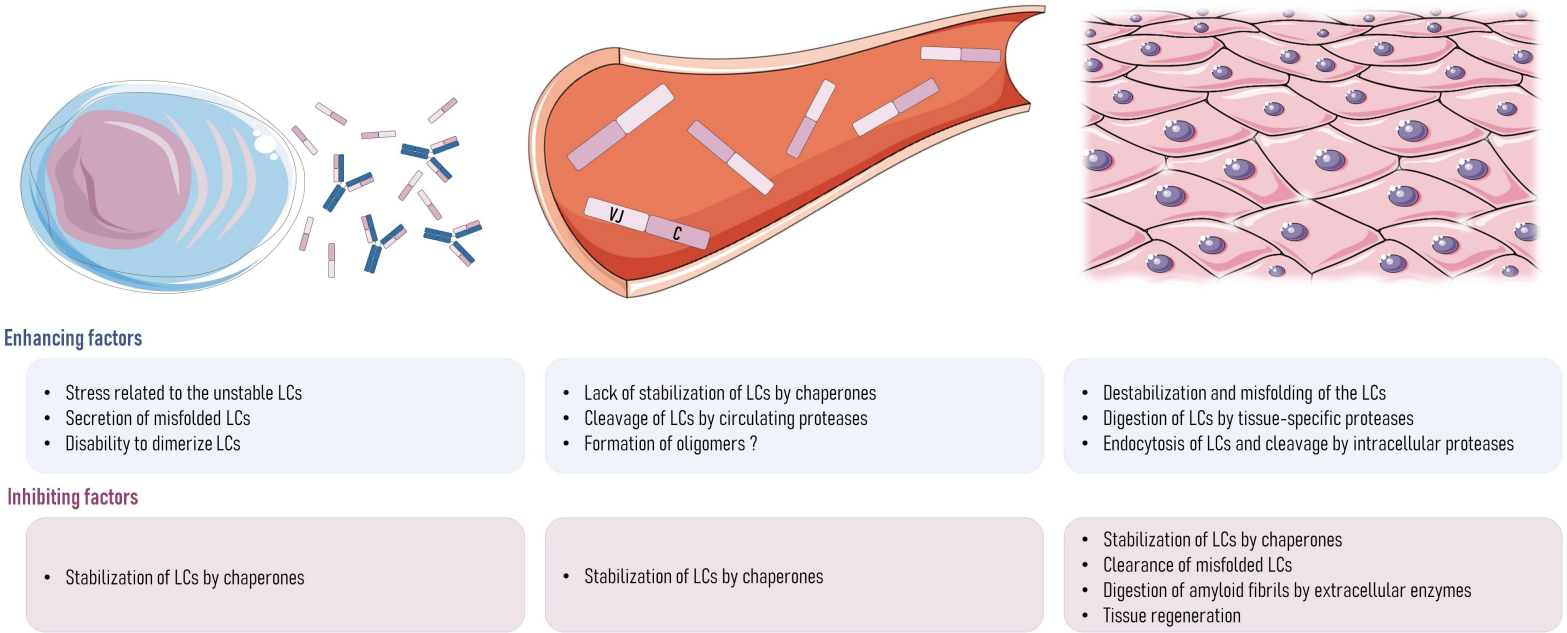
Hypotheses about what could be wrong with AL amyloidosis in rodents. In plasma cells, the stress of secreting a large amount of unstable pathogenic LCs may lead to a dysfunction of the protein-folding regulatory controls and the subsequent secretion of misfolded or unfolded LCs or the secretion of monomers instead of dimers. Once that LCs are in the bloodstream, LC structure could be destabilized by the lack of interaction with chaperones, transient changes in LC microenvironment (pH or presence of destabilizing molecules), or the initial cleavage of the LCs by proteases, releasing the amyloidogenic parts of the LC (VL). In tissues, there could also be the destabilization and misfolding of the LC structure by microenvironment changes, or their cleavage by extracellular proteases, such as the metalloproteases family or other proteases like plasmin. If LCs were endocytosed by tissue cells, cleavage could happen inside the cells, and this could involve any proteolytic enzyme, such as cathepsins. Any of these events could lead to a lack of stability of LCs in AL amyloidosis patients, which could lead to the initiation of the aggregation process and the deposit of fibrils into the organs. Any of them could also be different in rodents, explaining the absence of spontaneous amyloidosis even when high amounts of free LCs are produced. In addition to the short life span of mice that could account for the absence of spontaneous AL amyloidosis (lag phase lasting many years in humans), any of the mechanisms cited above could be different in rodents, explaining their apparent resistance to AL amyloidosis. It could also be explained by a more efficient clearance mechanism of misfolded proteins in mice leading to the rapid elimination of amyloid species before they reach the elongation phase (97). In the same way, faster tissue regeneration in mice could also explain their resistance to amyloidosis. LCs, light chains.

A similar strategy was used to generate an AL amyloidosis mouse model (89, 96). In that case, the full λ LC (VL and CL domains) from a patient suffering AL amyloidosis with cardiac and renal involvements, biopsy-proven amyloid deposits in the kidney, and low FLC levels (<100 mg/L) was introduced by knock-in in the Jκ locus of the mouse LCs. The amyloidogenicity of this LC was confirmed by *in vitro* fibrilization experiments. Unfortunately, and despite the secretion of about 400 µg/ml of amyloidogenic free LCs, which is four times that observed in the patient, these mice never developed amyloid deposits spontaneously in any organ, even in old mice, and histological analyses did not show any sign of organ impairment. This is in stark contrast with other models of LC-related monoclonal diseases. Circulating LC levels in LCDD or Fanconi patients are usually higher than in AL patients, but the mice reproduced the diseases in a few weeks or months (87, 90). It means that in contrast to other LC-induced diseases, AL amyloidosis does not spontaneously develop due to the sole LC and likely requires additional events that remain to be determined (**Figure 3**). Mice are indeed known to be a poor model for spontaneously forming amyloidosis including systemic amyloidosis or Alzheimer’s disease (98, 99). One reason could be a more effective mechanism of clearance or a better chaperoning of misfolded proteins than in humans as discussed later in this review (**Figure 3**) (100, 101). However, another transgenic mouse model reproduced perfectly human lesions of AAPOA2 amyloidosis (102), and cardiac ATTR deposits can be induced in a transgenic model by overpassing the lag phase with a seeding strategy (103), although the animals did not seem to present any morbidity. This leads to the belief that the resistance to amyloid formation is not complete and could be likely overcome to induce AL amyloidosis. With the amyloid lag phase being a long process, we cannot exclude that the level of free LCs, despite being higher than in patients, would need to be further increased to form detectable amyloid deposits during the lifetime of the mice. We are currently working on strategies to further improve the production of pathogenic LCs (89). Since the level of circulating free LCs was far above that observed in previous transgenic models (including the zebrafish), we would have expected organ dysfunctions due to the direct toxicity of the soluble AL LCs. However, their follow-up years seem to indicate no differences in the life span when compared to control mice, and they do not present any signs of morbidity or organ dysfunction related to their pathogenic LC expression. More careful investigations have to be conducted at the cellular level, including ROS production, apoptosis, or proliferation, but we do not believe that such alterations, even at a low level, would not have affected the survival of the animals, especially considering that the cardiac regeneration in mammals is almost absent. This resistance to spontaneous amyloidosis in either AL or ATTR mice models, together with the apparent absence of morbidity in the later, raises the interesting possibility that amyloid formation is in fact directly linked to the cellular or tissue-based toxicity exerted by the amyloidogenic protein. The absence of cellular toxicity in mice, possibly due to a more effective clearance of misfolded proteins compared to humans, could thus prevent the creation of favorable conditions for the formation of amyloidosis.

Accordingly, recent preliminary data from our lab showed that similar to the ATTR transgenic model (103), seeding with injections of AL fibrils made up *in vitro* with the VL portion of the transgenic LCs can lead to amyloid deposits in organs of our AL transgenic mice, especially the heart, spleen, vessel walls, and, to a lesser extent, kidney (104). Although these results have to be confirmed in more mice and carefully interpreted, they could represent a breakthrough in the modeling of AL amyloidosis.

## Where are we and where are we going: Could experimental models help fill the gaps in AL amyloidosis?

Despite all the experimental studies on AL amyloidosis cited in this review, which have considerably improved our understanding of the disease, any current publication on the subject will continue to start with this disappointing statement: the mechanisms underlying the development of amyloidosis in patients are still incompletely understood. The main barrier to better delineating common mechanisms of amyloidogenesis is the variability of the LCs, making each LC a unique molecular model. The scientific community also needs to solve the discrepancies observed between experimental approaches (amyloid species used, concentrations, and human or recombinant origin) and models (type of cells and animals). One of the main discrepancies is the difficulty to form amyloid fibrils *in vivo* when compared to those obtained easily *in vitro*. There is a clear gap in our understanding of the enhancing or inhibiting role of the microenvironment in *in vivo* amyloidogenesis (i.e., cellular interactions, glycosaminoglycans, local pH, and extracellular matrix composition) (51, 105–108) that cannot be solved *in vitro*, at least on simple cellular models, and requires a still missing reliable animal model (89). Such a model should provide clues about the involvement of LC proteolysis in the process of fibril formation (**Figure 3**). Whether before or after deposits form, the role of proteases in the degradation of the non-fibrillary part of the LCs has to be understood since it could not only explain discrepancies between mice and humans in their ability to form AL amyloidosis but also help discover enhancing or protecting factors that could become new targets for therapies (**Figure 3**). Increased activity of extracellular matrix metalloproteinases (MMPs) has been highlighted in cardiac and glomerular deposits in patients (109–112), but no study has demonstrated their specific involvement in amyloidogenesis or LC degradation. Proteases have been recently involved in TTR amyloidosis first *in vitro* (113) and then confirmed in a mouse model of ATTR (103). The authors showed that the plasmin pathway was activated in cardiac tissue and would be involved in the release of the amyloidogenic portion of TTR. Whether a similar process could be involved in AL amyloidosis remains to be determined. However, the spectrum of possible involved proteases is wide and includes intracellular proteases since LCs can also be internalized by many cell types (31, 51, 52, 56–58, 114).

We also have to consider that LC modifications, leading to their propensity to aggregate, could happen before they reached their final organ destination or even before their secretion by plasma cells. Since growing evidence showed that unstable, pathogenic LCs induce molecular stress in plasma cells (83, 90), their protein quality control, regulated by numerous ER-resistant chaperones, could be overwhelmed, leading to unfolded or misfolded LCs released in the bloodstream. Such proteins could then have a higher propensity to aggregate in tissues (115). Here, we reach another limit of AL modeling since a plasma cell quality control defect could also be due to the molecular abnormalities underlying the hematological disorder (i.e., translocations and mutations leading to the monoclonal proliferation) and then would be hardly reproducible in animal models with normal mouse plasma cells. Finally, extracellular chaperones, produced by plasma cells or any other cells, could also play a role. Chaperones, such as ERDJ3, are guardians of the proteins’ integrity: they can bind to misfolded proteins in the ER and remain associated after their secretion to prevent aggregation (116). Any defect in ERDJ3 production could lead to a loss of protection against circulating unfolded proteins and favor amyloidogenesis. Another chaperone, clusterin, which is currently associated with amyloid fibrils, is significantly decreased in patients with cardiac amyloidosis, which suggests that it could also play a role by protecting from amyloidogenesis (117). All these chaperones are highly conserved in mammals, but discreet differences in structure or concentration between humans and mice could account for the difficulties to reproduce spontaneous amyloidosis later (99). As an example, SAP, invariably found in amyloid deposits, was shown to protect fibrils from degradation by proteases (118), but human SAP binds much more avidly and is more abundant in human amyloid fibrils than in mice (119). Understanding these differences between rodents and humans would likely help decipher the last secrets of AL amyloidosis and design new therapeutic approaches.

## Conclusion

Studying AL amyloidosis is like trying to put together the pieces of a giant jigsaw puzzle in which a few pieces are still missing. Finding these missing pieces could help to finally understand the mechanism that allows the transition from a structure that should protect us to its pathogenic deposing-state counterpart that destroys the organs. It is even more true in AL than in any other amyloidosis since the diversity of the pathogenic LCs together with the variety of the clinical presentations greatly complicates this process. Data obtained from experimental models reflect this diversity, and *in vivo* models are needed to validate the questions raised by *in vitro* experiments. They have already been useful for testing new therapeutic approaches and launching debates about the intrinsic toxicity of the LCs. Whether or not *in vivo* models of AL amyloidosis could be game changers for understanding the disease remains to be confirmed, but we think that the efforts of the amyloidosis scientific community trying to develop a reliable AL amyloidosis model will one day be rewarded.

## Author contributions

GM-R and CS contributed to researching data for the article, writing, reviewing and editing the article before submission. SB contributed to researching data for the article. All authors contributed to the article and approved the submitted version.

## Funding

This work was supported by grants from Agence National de la Recherche (#ANR-21-CE17-0040-01), Fondation pour la Recherche Médicale (# FRM-EQU202203014615), Fondation Française pour la recherche sur le Myelome et les Gammapathies monoclonales (FFRMG) and Ligue nationale contre le cancer. GM-R is funded by fellowships from Région Nouvelle Aquitaine. SB is supported by Centre Hospitalier Universitaire Dupuytren Limoges and Plan National Maladies Rares.

## Conflict of interest

The authors declare that the research was conducted in the absence of any commercial or financial relationships that could be construed as a potential conflict of interest.

## Publisher’s note

All claims expressed in this article are solely those of the authors and do not necessarily represent those of their affiliated organizations, or those of the publisher, the editors and the reviewers. Any product that may be evaluated in this article, or claim that may be made by its manufacturer, is not guaranteed or endorsed by the publisher.

## Abbreviations

Ig, immunoglobulin; AL, light chain amyloidosis; LC, immunoglobulin light chains; VL, variable domain; CL, constant domain; Cryo-EM, cryogenic electron microscopy; SAP, serum amyloid P component; GAG, glycosaminoglycan; ApoE, apolipoprotein E; ROS, reactive oxygen species; NT-proBNP, N-terminal pro-B-type natriuretic peptide; SORL1, sortilin-related receptor 1; ThT, thioflavin T; FLC, free light chains; MGRS, monoclonal gammopathy of renal significance; LCDD, light chain deposition disease; LCTP, light chain proximal tubulopathy; PCs, plasma cells; LMP2A, latent membrane protein 2A; HCDD, heavy chain deposition disease; AAPOA2, amyloid ApoA2; ATTR, amyloid transthyretin; TTR, transthyretin; MMP, metalloproteinase.
